# Pervasive but Neglected: A Perspective on COVID-19-Associated Pulmonary Mold Infections Among Mechanically Ventilated COVID-19 Patients

**DOI:** 10.3389/fmed.2021.649675

**Published:** 2021-06-14

**Authors:** Mona Ghazanfari, Amir Arastehfar, Lotfollah Davoodi, Jamshid Yazdani Charati, Maryam Moazeni, Mahdi Abastabar, Iman Haghani, Roghayeh Mirzakhani, Sabah Mayahi, Wenjie Fang, Wanqing Liao, M. Hong Nguyen, David S. Perlin, Martin Hoenigl, Weihua Pan, Mohammad T. Hedayati

**Affiliations:** ^1^Invasive Fungi Research Center, Communicable Diseases Institute, Mazandaran University of Medical Sciences, Sari, Iran; ^2^Department of Medical Mycology, School of Medicine, Mazandaran University of Medical Sciences, Sari, Iran; ^3^Center for Discovery and Innovation, Hackensack Meridian Health, Nutley, NJ, United States; ^4^Antimicrobial Resistance Research Center/Department of Infectious Diseases, Faculty of Medicine, Mazandaran University of Medical Sciences, Sari, Iran; ^5^Department of Biostatistics, Faculty of Health, Mazandaran University of Medical Sciences, Sari, Iran; ^6^Medical Mycology, Shanghai Changzheng Hospital, Second Military Medical University, Shanghai, China; ^7^Department of Medicine, University of Pittsburgh, Pittsburgh, PA, United States; ^8^Clinical and Translational Fungal-Working Group, University of California, San Diego, La Jolla, CA, United States; ^9^Section of Infectious Diseases and Tropical Medicine, Department of Internal Medicine, Medical University of Graz, Graz, Austria; ^10^Division of Infectious Diseases and Global Public Health, Department of Medicine, University of California, San Diego, San Diego, CA, United States

**Keywords:** COVID-19-associated pulmonary mold infections, mechanically ventilation, galactomannan, *Aspergillus*, *Fusarium*

## Abstract

**Background:** Recent studies from multiple countries have shown a high prevalence of coronavirus disease 2019 (COVID-19)-associated pulmonary aspergillosis (CAPA) among severely ill patients. Despite providing valuable insight into the clinical management of CAPA, large-scale prospective studies are limited. Here, we report on one of the largest multicenter epidemiological studies to explore the clinical features and prevalence of COVID-19-associated pulmonary mold infections (CAPMIs) among mechanically ventilated patients.

**Methods:** Bronchoalveolar lavage (BAL) and serum samples were collected for culture, galactomannan (GM), and β-D-glucan (BDG) testing. Patients were classified as probable CAPMI based on the presence of host factors, radiological findings, and mycological criteria.

**Results:** During the study period, 302 COVID-19 patients were admitted to intensive care units (ICUs), among whom 105 were mechanically ventilated for ≥4 days. Probable CAPMI was observed among 38% of patients (40/105), among whom BAL culture of 29 patients turned positive for molds, while galactomannan testing on BAL (GM index ≥1) and serum (GM index >0.5) samples were positive for 60% (24/40) and 37.5% (15/39) of patients, respectively. *Aspergillus* (22/29; 75.8%) and *Fusarium* (6/29; 20.6%) constituted 96.5% of the molds isolated. *Diaporthe foeniculina* was isolated from a COVID-19 patient. None of the patients who presented with CAPMI were treated with antifungal drugs.

**Conclusion:** Despite being prevalent, the absence of appropriate antifungal treatment highlights that CAPMI is a neglected complication among mechanically ventilated COVID-19 patients admitted to ICUs. CAPMI can be caused by species other than *Aspergillus*.

## Introduction

Coronavirus disease 2019 (COVID-19) has hit almost every country in the world (1). The global mortality rate caused by COVID-19 has been ~3%. Numerous studies have shown that patients with underlying conditions, such as hypertension, obesity, diabetes, and higher age, are predisposed to develop more severe disease including acute respiratory distress syndrome (ARDS), which carries a higher mortality rate (2–6). Apart from these underlying conditions and the multiple organ damage exerted by severe acute respiratory syndrome coronavirus 2 (SARS-CoV-2), the presence of secondary infections, including bacterial and fungal infections, may contribute to a higher mortality (4). Therefore, rapid diagnosis and prompt initiation of antibiotic and/or antifungal therapies in the form of targeted treatment are of paramount importance to improve outcome. Fulfilling these goals, however, requires conducting prospective and large-scale epidemiological studies to establish appropriate therapeutic regimens as well as identify the most sensitive and specific diagnostic tools.

Invasive pulmonary aspergillosis (IPA), most commonly caused by *Aspergillus fumigatus*, is a recognized complication of severe influenza, especially among those patients with ARDS receiving corticosteroid therapy (7, 8). The mortality rate associated with influenza-associated IPA is high, even among patients with no known immunosuppression (7). IPA also has recently been linked to patients with severe COVID-19 requiring care in intensive care units (ICUs) (6, 9). There was evidence in some studies that antifungal therapy in these patients might improve outcome (6, 10). Prospective, large-scale studies, however, involving a large number of severely ill mechanically ventilated COVID-19 patients are limited to a recent study from the United Kingdom (10).

Iran has been ranked as the sixth country in terms of COVID-19 cases with a mortality rate two times higher than that reported globally (https://covid19.who.int/). Northern regions of Iran were among the initial epicenters of COVID-19. The upward and persisting trend of COVID-19 in this region has caused ~50,000 deaths since the beginning of the epidemic. Prospective multicenter studies assessing the prevalence of COVID-19-associated pulmonary mold infections (CAPMIs) from a large number of COVID-19 patients, especially from epicenters, are lacking in Iran. Herein, we explored the clinical features of CAPMI cases among 105 severely ill, mechanically ventilated COVID-19 cases in Iran.

## Materials and Methods

### Patients and Sample Collection

This cross-sectional study started on May 1 and ended on September 30, 2020. COVID-19 was diagnosed using real-time PCR and chest tomography (CT) images. Mechanically ventilated and PCR-confirmed COVID-19 patients with ARDS admitted to ICUs of three educational hospitals of Mazandaran University of Medical Sciences located in two cities of Mazandaran Province were recruited in our study. These centers are among the main centers for admission of severely ill COVID-19 cases from across the Mazandaran province.

In the study protocol that was approved by the institutional review board (IRB), bronchoalveolar lavage (BAL) sampling was set to occur 3–4 days after the start of mechanical ventilation in COVID-19 patients. Moreover, blood samples (5 ml) were collected from patients recruited in our study, and serum samples were obtained and stored at −80°C for future use. This study was approved by the ethics committee of the Mazandaran University of Medical Sciences (Code: IR.MAZUMS.REC.1399.7697).

### Sample Processing and Mold Isolate Identification

BAL samples were centrifuged for 10 min at 3,000 rpm. The supernatant was stored at −80°C for future uses. The sediment was inoculated on Sabouraud dextrose agar (QUELAB, Montreal, Quebec, Canada) supplemented with chloramphenicol (Sigma) (SC) and incubated at 27°C for 5–7 days and examined on a daily basis for fungal growth.

Mold colonies were subcultured onto SC and identified by morphological characteristics. Species-level identification involved sequencing beta-tubulin and ITS loci as described previously (11). The partial DNA sequence data from both genes were used as the BLAST query against three Web-accessible databases, those of the Centraalbureau voor Schimmelcultures (CBS-KNAW) Fungal Biodiversity Center, Utrecht, Netherlands (http://www.cbs.knaw.nl); the National Center for Biotechnology Information, Bethesda, MD (http://www.ncbi.nlm.nih.gov); and Fuasrium- ID (http://isolate.fusariumdb.org/blast.php).

### Galactomannan and β-D-Glucan Assay

Serum and BAL galactomannan (GM) antigen was measured using Dynamiker *Aspergillus* Galactomannan Assay DNK-1402-1 (Dynamiker Biotechnology, Tianjin, China) following manufacturer's instructions for quantitative detection. Samples were tested in duplicate, and the mean value was used for interpretation. Serum GM index >0.5 and BAL GM index ≥1.0 were considered positive results.

Serum β-D-glucan (BDG) was detected using the Dynamiker^®^ Fungus (1–3)-β-D-Glucan assay (Dynamiker Biotechnology, Tianjin, China) following manufacturer's instructions, with a positivity threshold >95 pg/ml. Samples were tested in duplicate, and the mean value was used for interpretation.

### COVID-19-Associated Pulmonary Mold Infection Definition

To date, the case definition of CAPMI has not been standardized. In this current study, we used a combination of the definition proposed by Koehler et al. (12) and Verweij et al. (13) for diagnosis of COVID-19-associated pulmonary aspergillosis (CAPA). Patients were classified as probable CAPMI based on the presence of host factors (requiring ICU admission for respiratory distress with a positive SARS-CoV-2 PCR temporally related to ICU admission), radiological factors [pulmonary infiltrate, preferably documented by chest CT or cavitating infiltrate (not attributed to another cause)], and mycological criteria. The mycological criteria were defined as the presence of at least one of the following: serum GM index >0.5 or BAL GM index ≥1.0 or positive respiratory specimen culture for mold pathogen. Positive BDG in serum was considered a supplementary test for diagnosis of CAPMI. If respiratory culture grows *Aspergillus* spp. or *Fusarium* spp., patients fulfilling the above criteria will be classified as CAPA or CAPfusariosis, respectively.

### Antifungal Susceptibility Testing

Isolates of *Aspergillus* and *Fusarium* species were subcultured on potato dextrose agar (PDA) for 7 days at 30°C. *In vitro* susceptibility testing was performed according to the Clinical & Laboratory Standards Institute (CLSI) M38-A2 guidelines (14). Antifungal agents included were as follows: amphotericin B (AMB; Bristol-Myers Squibb Co., Princeton, NJ, USA); itraconazole (ITR; Janssen Pharmaceutica N.V., Beerse, Belgium); voriconazole (VOR; Pfizer, Sandwich, United Kingdom); ravuconazole (RAV; Sigma-Aldrich, Steinheim, Germany); posaconazole (POS; Merck Sharp & Dohme BV, Haarlem, Netherlands); isavuconazole (ISA; Basilea Pharmaceuticals, Basel, Switzerland); and anidulafungin (ANF; Pfizer, Sandwich, United Kingdom).

Typically, final concentrations of the antifungal agents ranged from 0.016 to 16 μg/ml for AMB, VOR, ITR, RAV, and POS; and from 0.008 to 8 μg/ml for the echinocandins. *Candida parapsilosis* (ATCC 22019) and *Candida krusei* (ATCC 6258) were also used as quality control strains. The minimum inhibitory concentrations (MICs) were recorded visually with complete inhibition of growth following 48-h incubation at 37°C, while minimum effective concentrations (MECs) were read for echinocandins microscopically following 48–72-h incubation at 37°C.

### Statistical Analysis

The demographic and clinical characteristics of patients were described using mean and median frequencies and percentage. Mann–Whitney or Student's *t*-test and chi-square test or Fisher's exact test were used to compare factors between the two groups (CAPMI and non-CAPMI). The analyses were performed using the Statistical Package for Social Sciences (SPSS) software version 25.0. *P* < 0.05 was considered a statistically significant difference.

## Results

### Patients' Characteristics

**Figure 1:**
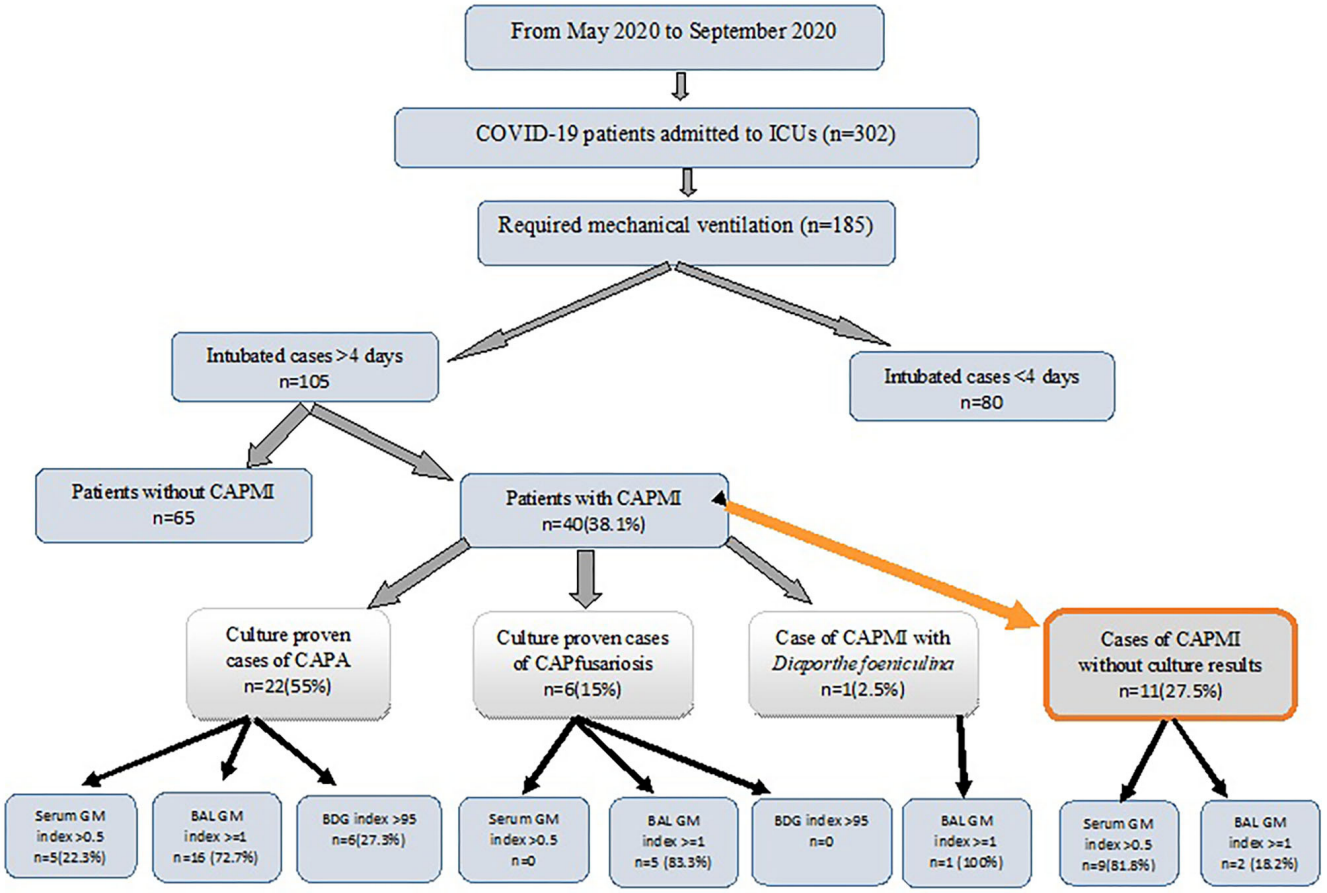
The diagnostic breakdown of the study population. ICU, intensive care unit; BAL, bronchoalveolar lavage; GM, Galactomannan; BDG, β-D-glucan test; CAPMI, COVID-19 associated pulmonary mold infections; CAPA, COVID-19 associated pulmonary aspergillosis; CAPfusariosis, COVID-19 associated pulmonary fusariosis.

Over the study period, 302 patients were admitted to ICUs, of whom 185 required mechanical ventilation. Eighty patients were extubated within 4 days and were excluded from the analysis. The remaining 105 patients who required mechanical ventilation for ≥4 days formed the basis for this study (Figure 1). Of 105 patients, 58 (55.2%) were male. The demographic and clinical characteristics are presented in Table 1. The patients' ages ranged from 25 to 95 years with a mean of 65.2 years. Of 105 patients, 14 did not have any underlying conditions, while underlying conditions were noted in 91 cases, among which hypertension (48/105; 45.7%), ischemic heart disease (41/105; 39%), and diabetes mellitus (40/105; 38%) were the most common.

**Table 1:** Demographic, underlying conditions and clinical characteristics of critically ill COVID-19 patients with and without COVID-19 associated pulmonary mold infection.

		**Population**			
		**All mechanically ventilated patients (*n* = 105)**	**Probable CAPMI (*n* = 40)**	**No CAPMI (*n* = 65)**	***P*-value**
Median Age		65	67	65	0.29
Male/female		58/47	19/21	39/26	0.15
Mortality rate^*^		102/103 (97.1%)	37/40 (92.5%)	65/65 (100%)	0.053
ICU stay before sample collection (mean- median/days)		10.50–9.00	9.35–7.00	6.53–6.00	0.04
Underlying conditions *n* (%)	Smoking	22 (20.9%)	2 (5%)	20 (30.8%)	0.002
	HTN	48 (45.7%)	16 (40%)	32 (49.2%)	0.356
	DM	40 (38.1%)	11 (27.5%)	29 (44.6%)	0.079
	IHD	41 (39.0%)	14 (35%)	27 (41.5%)	0.505
	Steroid use	5 (4.8%)	3 (7.5%)	2 (3.1%)	0.301
	Asthma	6 (5.7%)	3 (7.5%)	3 (4.6%)	0.536
	Previous TB	5 (4.8%)	2 (5%)	3 (4.6%)	0.928
	CKD	7 (6.7%)	3 (7.5%)	4 (6.1%)	0.788
	CML	1	1 (2.5%)	–	–
	Renal failure	1	1 (2.5%)	–	–
	Obesity	4 (3.8%)	4 (10%)	–	–
	Hypothyroidism	1	1(2.5%)	–	–
	Intestinal Cancer	1	1 (2.5%)	–	–
	HIV	1	1 (2.5%)	–	–
	Nephrolithiasis	1	–	1 (1.5%)	–
	Nothing	14 (13.3%)	1 (2.5%)	13 (20%)	–
Corticosteroids use (*n*/*N*, %)	Pre– Hospital	3/105 (2.8%)	2/40 (5%)	1/65 (1.5%)	0.3
	In Hospital	105/105 (100%)	40/40 (100%)	65/65 (100%)	–
Steroid duration before sample collection (mean- median/days)		8.50–8.00	5.55–5.00	4.52–4.00	0.056
**Mycology**					
BDG serum Mean Concentration [pg/ml, (95% CI)]		51.09 (45.1–57.06)	70.06 (60.74–79.38)	39.15 (32.9–45.4)	0.001
BDG serum sample positivity [*n*/*N*, (%, 95% CI)]		6/101 (5.9%, 1.3–10.49)	6/40 (15%, 3.9–26.1)	–	–
GM– BAL Mean		0.8 (0.6–1.0)	1.53 (1.12–1.95)	0.34 (0.28–0.39)	0.001
GM-BAL patient positivity rate [*n*/*N* (%, 95% CI)]		24/105 (22.9%, 14.86–30.94)	24/40 (60.0%,44.82–75.18)	–	–
GM -Serum Mean		0.37 (0.32–0.42)	0.52 (0.39–0.64)	0.28 (0.26–0.30)	0.001
GM -Serum patient positivity rate [*n*/*N* (%, 95% CI)]		15/101 (14.8%, 8.68–24.48)	15/39 (38.5%, 24.82–55.18)	–	–
**Radiological findings**					
Bilateral multifocal peripheral patchy GGO		77 (73.3%)	28 (70%)	49 (75.4%)	0.55
Bilateral multilobar GGO + consolidation extension 25–50%		26 (24.7%)	10 (25%)	16 (24.6%)	0.97
Bilateral multifocal peripheral patchy GGO + Cavity		2 (2.0%)	2 (5%)	–	–
**Laboratory finding and signs**					
Total Leucocytes (median)		12.5	14.13	13.85	0.071
Cough *n* (%)		104 (99)	40 (100)	64 (98.5)	0.62^*^
Headache *n* (%)		43 (41)	19 (47.5)	24 (36.9)	0.28
Nausea *n* (%)		24 (22.9)	9 (22.5)	15 (23.1)	0.94
Lethargy *n* (%)		72 (68.6)	28 (70)	44 (67.7)	0.8
Recurrence of the COVID- 19 n(%)		5(4.8)	2(5)	3(4.6)	0.93

*GM, Galactomannan; BDG, (1–3)-β-D-Glucan; HTN, Hypertension; DM, Diabetes mellitus; CML, Chronic myelogenous leukemia; IHD, Ischemic heart disease; CKD, Chronic kidney disease; GGO, ground glass opacity*.

* *Overall mortality rate of hospitalized COVID-19 was 33.8% (102/302) among all ICU patients with/without being mechanically ventilated, while it reached 55.1% (102/185) for those who underwent mechanical ventilation*.

As per institutional standard of care at the time the study was conducted, all 105 COVID-19 patients received antimicrobial therapy comprising of ceftriaxone (1 g IV once a day or in equally divided doses twice a day) and azithromycin (500 mg once per day for 5 days). Upon ICU admission, therapy was empirically broadened to vancomycin (1 g q12 h IV; total daily dose = 2.0 g) and imipenem (IPM; 500 mg q6 h IV; total daily dose = 2.0 g) due to high local rates of methicillin-resistant *Staphylococcus aureus* (MRSA) and multiresistant Gram-negative pathogens.

Three patients were receiving corticosteroid (prednisone: tablet, 5 mg/day) prior to hospitalization for control of asthma (Table 1). As per institutional standard of care (criteria for steroid use), all 105 patients also received dexamethasone (8 mg PO/IV qDay) after hospitalization and also during ICU stay. The mean and median duration of ICU stay was 10.5 and 9 days, respectively. The overall mortality rate of hospitalized COVID-19 cases in our study was 33.8% (102/302) among all ICU patients with/without being mechanically ventilated, while it reached 55.1% (102/185) for those who underwent mechanical ventilation.

### Mycological Findings in COVID-19-Associated Pulmonary Mold Infection Patients

Forty patients (38.1%) fulfilled our definition of probable CAPMI. None had proven CAPMI. Twenty-nine (27.6%) had a positive BAL culture. CAPA was identified in 22 (75.9%) patients, of which *Aspergillus flavus* (14/22, 63.6%) was the most common agent, followed by *A. fumigatus* and *Aspergillus japonicus* (3/22 and 13.6% each) and *Aspergillus niger* (2/22, 9.1%). In patients with CAPfusariosis (6/29, 20.7%), *Fusarium incarnatum* (50%) was the most common followed by *Fusarium fujikuroi* (16.6%) and *Fusarium equiseti* and *Fusarium solani* (16.6% each). In one case of CAPMI, *Diaporthe foeniculina* was isolated (Table 2).

**Table 2:** Clinical features of COVID-19 patients with probable invasive mold infections.

**Cases**	**Age/sex**	**Underlying conditions**	**Radiological findings**	Culture results (no. of isolates)^*^	**GM results**		**BDG results**	**ICU duration**	**Time of ICU admission to mycological positivity**	**Time to death after positivity**	**Outcome**
					**BAL**	**Serum**					
1	63/Female	HTN - DM	Bilateral multifocal peripheral patchy GGO	*F. fujikuroi* (4)	0.4	0.3	92.1	5	4	1	Died
2	67/Male	Athma	Bilateral multilobar GGO and consolidation extension 25–50%	*A. fumigatus* (3)	0.2	0.4	77.9	7	4	3	Died
3	61/Male	CML	Bilateral multifocal peripheral patchy GGO	*A. flavus* (4)	0.4	0.4	6.9	10	6	4	Died
4	48/Male	Athma	Bilateral multifocal peripheral patchy GGO	*A. fumigatus* (4)	1.1	0.2	105.6	8	5	3	Died
5	84/Male	Smoker- HTN-Renal failare	Bilateral multilobar GGO and consolidation extension 25–50%	*F. incarnatum* (5)	2.3	0.2	72.7	8	5	3	Died
6	77/Female	HTN-IHD	Bilateral multilobar GGO and consolidation extension 25–50%	*F. equiseti* (4)	3.7	0.3	80.4	8	5	3	Died
7	82/Male	Smoker	Bilateral multifocal peripheral patchy GGO	*A. japonicus* (5)	1.5	0.4	98.6	10	6	4	Died
8	74/Female	HTN-DM	Bilateral multifocal peripheral patchy GGO	*A. flavus* (2)	1.2	0.2	102.7	12	7	5	Died
9	70/Male	HTN-DM-IHD	Bilateral multifocal peripheral patchy GGO	*F. incarnatum* (2)	2.9	0.2	78.6	9	7	2	Died
10	75/Female	HTN-DM-IHD	Bilateral multilobar GGO and consolidation extension 25–50%	*A. flavus (1)*	4.6	0.9	50.6	15	10	5	Died
11	84/Female	CKD –DM-HTN	Bilateral multilobar GGO and consolidation extension 25–50%	*A. flavus* (2)	1.9	0.3	79.6	20	15	5	Died
12	67/Female	DM- IHD -CKD -HTN	Bilateral multifocal peripheral patchy GGO	*Diaporthe foeniculina* (2)	2.1	0.2	83.7	9	7	2	Died
13	67/Female	DM –IHD–HTN	Bilateral multifocal peripheral patchy GGO	*A. flavus* (3)	1.6	0.6	93.9	8	5	3	Died
14	59/Female	IHD	Bilateral multifocal peripheral patchy GGO	*F. incarnatum* (2)	2.8	0.2	89.7	9	7	2	Died
15	63/Female	DM	Bilateral multifocal peripheral patchy GGO	*A. fumigatus* (4)	1.4	0.4	24.8	15	9	5	Died
16	71/Female	CKD –IHD- HTN	Bilateral multifocal peripheral patchy GGO	*A. japonicus* (2)	4.4	0.6	86.9	9	7	2	Died
17	76/Male	IHD -HTN	Bilateral multifocal peripheral patchy GGO	*A. flavus* (2)	1.6	0.5	94.6	30	25	5	Died
18	62/Female	DM -Obesity	Bilateral multilobar GGO and consolidation extension 25–50%	*A. niger* (3)	4.6	1.1	110.5	27	24	3	Died
19	68/Female	IHD -HTN	Bilateral multifocal peripheral patchy GGO	*A. flavus* (1)	1.9	2.4	106.4	4	3	1	Died
20	49/Female	Obesity	Bilateral multifocal peripheral patchy GGO	*F. solani* (2)	2.8	0.4	89.0	7	4	3	Died
21	62/Male	Previous TB	Bilateral multifocal peripheral patchy GGO + Cavity	*A. flavus* (2)	1.0	0.3	75.2	9	5	4	Died
22	75/Male	IHD -HTN	Bilateral multifocal peripheral patchy GGO	*A. flavus* (1)	3.5	0.5	42.9	11	8	3	Died
23	74/Male	IHD –HTN-asthma	Bilateral multifocal peripheral patchy GGO	*A. flavus* (3)	1.3	0.3	91.4	10	8	2	Died
24	52/Female	IHD-HTN -DM	Bilateral multilobar GGO and consolidation extension 25–50%	*A. flavus* (2)	0.5	0.9	115.9	7	4	3	Died
25	40/Male	Previous TB	Bilateral multilobar GGO and consolidation extension 25–50%	*A. niger* (4)	0.8	0.4	88.5	10	7	3	Died
26	59/Female	IHD -Obesity	Bilateral multifocal peripheral patchy GGO	*A. flavus* (2)	2.0	0.4	75.9	7	5	2	Died
27	59/Female	IHD -HTN	Bilateral multifocal peripheral patchy GGO	*A. japonicus* (1)	0.3	0.3	85.9	7	5	2	Died
28	62/Female	DM - Hypothyroidism -Obesity- Intestinal cancer- using long term corticosteroid	Bilateral multifocal peripheral patchy GGO	*A. flavus* (2)	0.3	0.2	65.9	60	53	–	Survived
29	67/Male	Nothing	Bilateral multifocal peripheral patchy GGO + Cavity	*A. flavus* (4)	4.7	–	ND	8	6	–	Survived
30	59/Female	Nothing	Bilateral multilobar GGO and consolidation extension 25–50%	–	0.9	0.8	18.5	7	4	3	Died
31	89/Male	HTN- Athma	Bilateral multifocal peripheral patchy GGO	–	0.5	0.7	27.5	12	8	4	Died
32	79/Female	HTN- DM	Bilateral multilobar GGO and consolidation extension 25–50%	–	0.4	0.6	66.2	4	3	1	Died
33	82/Male	HTN- DM	Bilateral multifocal peripheral patchy GGO	–	0.6	0.8	22.1	6	4	2	Died
34	72/Female	CKD- HTN	Bilateral multifocal peripheral patchy GGO	–	2.1	0.4	67.2	9	5	4	Died
35	72/Male	DM– IHD- HTN- smoker	Bilateral multifocal peripheral patchy GGO	–	0.4	0.6	46.7	21	15	6	Died
36	62/Male	HTN- IHD- DM	Bilateral multifocal peripheral patchy GGO	–	0.7	0.6	62.7	25	18	7	Died
37	65/Male	CKD- HTN- IHD	Bilateral multifocal peripheral patchy GGO	–	0.8	0.8	27.5	15	9	6	Died
38	73/Male	IGD- HTN- Smoker	Bilateral multifocal peripheral patchy GGO	–	1.2	0.2	70.4	12	8	4	Died
39	72/Female	DM	Bilateral multifocal peripheral patchy GGO	–	0.3	0.6	19.8	15	9	6	Died
40	25/Male	Previous TB- HIV	Bilateral multifocal peripheral patchy GGO	–	0.2	0.8	42.1	30	25	–	Survived

*GGO, Ground glass opacity; GM, Galactomannan; BDG, ß-D-Glucan test; F, Fusarium; A, Aspergillus; HTN < Hypertension; DM, Diabetes mellitus; CML, Chronic myelogenous leukemia; IHD, Ischemic heart disease; CKD, Chronic kidney disease; ND, not determined*.

* *Number of grown colonies from the culture medium*.

Of 40 patients with CAPMI, 17 (42.5%) had only one while 23 (57.5%) had more than one positive mycological findings. The patients with CAPMI were positive for GM BAL (GM index ≥1) and serum (GM index >0.5) in 24/40 (60%) and 15/39 (38.5%) cases, respectively. Serum BDG yielded positive results among 15.4% (6/39) of the CAPMI patients (Table 1). Of 29 patients with BAL-positive culture for mold, 22 (75.9%) had positive BAL GM index ≥1, including 16 patients with *Aspergillus* spp. growth, five with *Fusarium* species growth, and one with *D. foeniculina* growth. Of note, BAL and serum samples were positive in two and 10 patients for whom the BAL cultures were negative, respectively. Moreover, BDG testing was positive in six patients (>95 pg/ml), among whom five had positive GM on BAL, and three positive GM on serum (Table 2). More details are available in Figure 1.

### Clinical Characteristics of COVID-19-Associated Pulmonary Mold Infection Patients

At the time of ICU admission, all 105 included patients underwent a CT scan, and bilateral multifocal peripheral patchy ground-glass opacity (GGO) was observed in 77 (73.3%), bilateral multilobar GGO + consolidation extension 25–50% was observed in 26 (24.7%), and bilateral multifocal peripheral patchy GGO + cavity was observed in two (2.0%) patients (Table 1).

In the CT scan of 40 patients with CAPMI, bilateral multifocal peripheral patchy GGO was reported in 28 (70%), bilateral multilobar GGO + consolidation extension 25–50% was reported in 10 (25%), and bilateral multifocal peripheral patchy GGO + cavity was reported in two (5%) cases. There was no significant difference between the two groups of COVID-19 patients with CAPMI and those without CAPMI with respect to radiological findings (Table 1).

It was identified that the ICU stay duration was significantly higher among those with CAPMI (*p*-value: 0.04), while no difference was noted with regard to underlying conditions and mortality rate (Table 1). This difference was driven by a significant difference in ICU stay before sample collection was performed (defined as the time of having mycological test results) in CAPMI patients [mean = 9.35 (CI: 6.53–12.16) and median = 7.00 (IQR: 5.66–8.33) days] vs. in others [mean = 6.53 (CI: 6.05–7.01) and median = 6.0 (IQR: 5.47–6.52) days (*p*-value: 0.04)], as well as there was a significant difference in ICU stay after sample collection was performed [mean = 3.52 (CI: 3.01–4.03) and median = 3.0 (IQR: 2.44–3.55) days vs. mean = 2.47 (CI: 2.10–2.85) and median = 2.0 (IQR: 1.56–2.43) days (*p*-value: 0.004)]. The mean and median duration of steroid in included patients was 8.5 and 8.0, respectively. The mean and median duration of steroid from hospitalization before sample collection in CAPMI patients was 5.55 (CI: 4.45–6.64) and 5.0 (IQR: 3.45–6.54) days and those without mycological findings was 4.52 (CI: 3.94–5.10) and 4.0 (IQR: 3.28–4.71) days (*p-*value: 0.056), respectively.

Of three patients who were receiving prednisone prior to hospitalization for control of asthma, two showed CAPMI.

### Antifungal Susceptibility Testing

Table 3 represents the MICs of azole agents for the 29 mold strains. POS (MIC range 0.001–0.016 μg/ml, GM MIC 0.01 μg/ml) and AMB (MIC range 0.125–0.5 μg/ml, GM MIC 0.29 μg/ml) exhibited the lowest and the highest MICs against *A. flavus* isolates, respectively. The lowest and highest MIC_50_/MIC_90_ of *A. flavus* was observed against POS (0.016/0.016) and AMB (0.25/0.5). None of the *Aspergillus* species isolates showed MIC above epidemiologic cutoff value (ECV) against all tested antifungals. *F. equiseti* showed the highest MICs against VOR (16 μg/ml), ITR, POS, and ISA (8 μg/ml each). *F. solani* had MIC 8 μg/ml against VOR.

**Table 3:** *In-vitro* antifungal susceptibility profile of mold species isolated from COVID-19 patients against different antifungal agents.

**Isolated species**		***A. flavus***	***A. flavus***	***A. flavus***	***A. flavus***	***A. flavus***	***A. flavus***	***A. flavus***	***A. flavus***	***A. flavus***	***A. flavus***	***A. flavus***	***A. flavus***	***A. flavus***	***A. flavus***			***A. fumigatus***	***A. fumigatus***	***A. fumigatus***	***A. japonicus***	***A. japonicus***	***A. japonicus***	***A. niger***	***A. niger***	***Diaporthe foeniculina***	***F. fujikuroi***	***F. incarnatum***	***F. equiseti***	***F. incarnatum***	***F. incarnatum***	***F. solani***		
**Drugs**	**Range**	**MIC/MEC** **μg/mL**	**MIC/MEC** **μg/mL**	**MIC/MEC** **μg/mL**	**MIC/MEC** **μg/mL**	**MIC/MEC** **μg/mL**	**MIC/MEC** **μg/mL**	**MIC/MEC** **μg/mL**	**MIC/MEC** **μg/mL**	**MIC/MEC** **μg/mL**	**MIC/MEC** **μg/mL**	**MIC/MEC** **μg/mL**	**MIC/MEC** **μg/mL**	**MIC/MEC** **μg/mL**	**MIC/MEC** **μg/mL**	**MIC**_**50**_**/MIC**_**90**_	**Gmean**	**MIC/MEC** **μg/mL**	**MIC/MEC** **μg/mL**	**MIC/MEC** **μg/mL**	**MIC/MEC** **μg/mL**	**MIC/MEC** **μg/mL**	**MIC/MEC** **μg/mL**	**MIC/MEC** **μg/mL**	**MIC/MEC** **μg/mL**	**MIC/MEC** **μg/mL**	**MIC/MEC** **μg/mL**	**MIC/MEC** **μg/mL**	**MIC/MEC** **μg/mL**	**MIC/MEC** **μg/mL**	**MIC/MEC** **μg/mL**	**MIC/MEC** **μg/mL**	**MIC50/MIC90**	**Gmean**
AMB	0.032–8	0.25	0.125	0.25	0.25	0.25	0.5	0.5	0.25	0.25	0.5	0.25	0.5	0.25	0.25	0.25/0.5	0.29	0.25	0.063	0.063	0.25	0.25	0.032	0.032	0.5	2	1	0.125	4	0.25	0,032	8	0.25/1	0.27
ITR	0.016–8	0.032	0.032	0.032	0.032	0.032	0.032	0.032	0.063	0.016	0.032	0.032	0.0625	0.063	0.063	0.032/0.063	0.03	0.063	0.032	0.125	0.063	0.032	0.032	0.25	0.016	0.25	0.125	0.25	8	2	0.063	1	0.063/0.5	0.07
VOR	0.016–16	0.125	0.25	0.25	0.25	0.25	0.125	0.063	0.25	0.063	0.125	0.125	0.125	0.125	0.125	0.125/0.25	0.14	0.125	0.125	0.25	0.063	0.25	0.125	0.25	0.125	0.25	1	0.016	16	0.5	0.25	8	0.125/0.5	0.21
POS	0.016–8	0.016	0.016	0.016	0.016	0.016	0.016	0.016	0.016	0.016	0.016	0.016	0.016	0.016	0.016	0.016/0.016	0.01	0.016	0.016	0.016	0.016	0.016	0.016	0.016	0.016	0.063	0.25	0.016	8	0.016	0.016	0.32	0.016/0.032	0.02
RAV	0.016–4	0.016	0.016	0.016	0.016	0.016	0.016	0.063	0.032	0.016	0.016	0.016	0.032	0.125	0.032	0.016/0.063	0.02	0.032	0.063	0.063	0.016	0.016	0.016	0.063	0.032	4	4	0.016	2	0.25	0.063	0.25	0.032/0.5	0.05
ISA	0.016–8	4	0.063	0.063	0.016	0.032	0.016	0.016	0.063	0.016	0.016	0.016	0.063	0.125	0.063	0.032/0.125	0.04	0.125	0.063	0.125	0.063	0.063	0.032	0.125	0.016	0.063	1	0.032	8	0.125	0.032	0.25	0.063/0.5	0.07
ANF	O.008–4	0.008	0.008	0.008	0.008	0.008	0.008	0.008	0.008	0.008	0.008	0.008	0.008	0.008	0.008	0.008/0.008	0.008	0.008	0.008	0.008	0.008	0.008	0.008	0.008	0.008	0.008	1	4	2	4	4	1	0.008/2	0.02

## Discussion

This study presents a unique picture of CAPA and CAPMI in Iran when compared to other studies exploring the burden of CAPA among severely ill COVID-19 patients. Firstly, the species distribution was different from previously published cases, and we found *A. flavus* as the most prevalent mold species. Furthermore, for the first time, we observed species belonging to *Fusarium* and an environmental mold complicating COVID-19 in mechanically ventilated patients. It is worth noting that *A. flavus* was reported as the main contaminant species of *Aspergillus* in clinical and indoor hospital samples from Iran (15, 16). This may be due to climatic conditions or ongoing construction work in some of the participating hospitals. We showed that radiology was not beneficial to diagnose CAPA/CAPMI, as in our study, there was no significant radiologic difference between COVID-19 patients with CAPMI and those without CAPMI, while both culture and GM testing on BAL samples provided the most specific and sensitive tools.

In this study, we found that ~28% of the mechanically ventilated patients had positive culture for mold species, among whom almost 76% had positive GM on BAL, while BDG and GM using serum samples showed much lower sensitivity. These findings are consistent with those identified in other studies (17–21), in which GM and culture of BAL samples yielded the most accurate results. It is important to note that neither culture nor GM in BAL is a definitive method to diagnose pulmonary angio-invasive disease caused by fungi including *Aspergillus* species, which may also be positive in airway invasive disease and colonization. While antifungal treatment has been associated with improved survival in CAPA patients, some patients improved without antifungals, which argued against true invasive disease in these individuals (20). While histologically confirmed CAPA is increasingly reported, Flikweert et al. (22) did not find postmortem histological evidence of CAPA in six patients diagnosed with probable CAPA, although given the unreliable radiologic signs of CAPA and overlap with severe COVID-19, their method of ultrasound and CT-guided postmortem needle biopsies may have very well-randomly missed focal *Aspergillus* infection. Indeed, the most recent consensus statements of the European Confederation of Medical Mycology (ECMM) and International Society for Human & Animal Mycology (ISHAM) also have advocated the use of BAL and lower respiratory tract sampling while advocating against the utility of serum to be tested for both GM and BDG (12). Although tissue biopsy and the observation of invasion by septate hyphae are the gold standard and required for proven infection, tissue biopsies are rarely obtained *in vivo* due to the high contagious nature of SARS-CoV-2 (12). In our study, radiological imaging did not differ in those with CAPMI and those without, as suggested previously (12). Collectively, our experience suggests that BAL culture and BAL GM represent the most useful diagnostic tools, while testing serum using GM and BDG and radiological findings provide a lower level of assistance in the diagnosis of CAPA/CAPMI.

One of the main challenges in fungal infection diagnosis in ICU patients including COVID-19 is the lack of consensus on a diagnostic algorithm. Critically ill COVID-19 patients are usually without classical host factors (neutropenia, receipt of an allogeneic stem cell transplant) and clinical criteria (halo signs) for CAPMI, as defined by the European Organization for Research and Treatment of Cancer/Invasive Fungal Infections Cooperative Group and the National Institute of Allergy and Infectious Diseases Mycoses Study Group (EORTC/MSG), and will therefore not fulfill these criteria (23). Blot et al. (24) suggested a clinical algorithm to differentiate *Aspergillus* colonization from IPA in ICU patients without classical risk factors (AspICU algorithm) based on mycological criteria including detection of *Aspergillus* from respiratory specimens including BAL. More specifically for those with underlying acute viral diseases, Koehler et al. (12) and Verweij et al. (13) have recently proposed a diagnostic algorithm for CAPA and influenza-associated pulmonary aspergillosis, respectively. In these proposed criteria, patients with COVID-19 were divided into three groups of proven, probable, and possible invasive aspergillosis. Due to occupational safety and protection against virus transmission, classifying COVID-19 patients in proven IPA category is rarely possible in many institutions around the world, as local restrictions preclude biopsies and autopsies in COVID-19 patients. Using different algorithms for classification of fungal infections in COVID-19 patients may lead to different incidence rates of CAPMI. In the present study, two (cases 3 and 28) patients met the EORTC/MSG criteria (23), and the incidence rate of CAPMI was 27.6 and 38.1% according to the AspICU algorithm (24) and the combination definition proposed by Koehler et al. (12) and Verweij et al. (13), respectively. The prevalence of CAPA found in our study (22/105; 20.9%) is closer to that found in studies from Wales (14.1%) (10), Netherlands (19.4%) (19), and Pakistan (21.7%) (25) but lower than reported from Germany (26.3%) (21) and France (33.3%) (20). As for the species causing CAPMI, *A. flavus* constituted 63% of the *Aspergillus* isolates, which is in line with a recent systematic review on invasive aspergillosis in Iran, where *A. flavus* represented the most prevalent agent of IPA (26). This picture is entirely different from that of many other countries, where *A. fumigatus* has been shown to be the most prevalent agent of IPA and CAPA (10, 19–21). Importantly, we found that ~21% of the CAPMI cases were due to various species within the *Fusarium* genus, while to the best of our knowledge, there is only one previous case report of invasive pulmonary fusariosis due to *Fusarium proliferatum* in an immunocompetent critically ill patient with severe COVID-19 (27). Of note, five out of six fusariosis cases were identified *via* BAL GM testing; the cross-reactivity of GM with *Fusarium* spp. has been noted previously (28). Moreover, *Fusarium incarnatum* was the most common species among fusariosis cases (3/6), which is unlike previous studies that reported *F. solani* complex (comprising ~40–60% of infections), followed by *F. oxysporum* as the most common species in immunocompromised patients other than COVID-19 (29). Interestingly, we also found *D. foeniculina* in the BAL sample of one of the CAPMI cases, which is known for being a plant pathogen. This species has been rarely reported to cause infection in humans, and a few studies have shown a wide range of infections among transplant patients (30–32). Of note, the fact that our BAL sampling was restricted to a single sample obtained 3–4 days after initiation of mechanical ventilation may have resulted in underestimation of CAPMI.

Of particular concern is the lack of antifungal treatment among CAPA/CAPMI cases observed in this study, while it has been shown that CAPA cases receiving antifungal treatment had significantly lower mortality compared to those not treated (10). Moreover, the joint ECMM/ISHAM consensus statement (12) recommends using either VOR or ISA to treat CAPA. The underestimation of fungal infections, both superficial and invasive, is a well-known phenomenon in developing countries (33, 34), and increased awareness is needed.

In our study, the mean duration of steroid use from hospitalization to positive mycological tests in patients with CAPMI compared to those without was higher (5.55 vs. 4.52 days); however, the statistical significance was borderline (*p*-value: 0.056). These results may imply that the long stay in ICU and consequently the longer duration of steroids received by CAPMI patients predisposed them to opportunistic fungal infections. In addition, the widespread administration of different antibiotics among the patients recruited in this study and the ongoing construction work in some centers may have had an impact on the incidence of CAPMI in our study. However, the occurrence of CAPMI and construction requires more study to see if there is a link between the two.

The overall mortality rate of hospitalized COVID-19 cases in our study was 33.8% among all ICU patients with/without being mechanically ventilated, while it reached 55.1% for those who underwent mechanical ventilation. Since all patients recruited in our study were mechanically ventilated, no conclusions can be drawn from our data whether CAPMI is associated with higher overall mortality. On the other hand, the absence of a significant difference between CAPMI and non-CAPMI groups with respect to mortality may indicate fungal colonization rather than true invasive mold infection as has been suggested by Flikweert et al. (22).

In our study, only mechanically ventilated COVID-19 patients with ARDS were evaluated for fungal infections. Due to the special conditions of COVID-19 patients and the possibility of transmission, we were able to collect respiratory and blood samples only at one time point. Our study protocol also did not allow for obtaining biopsy or autopsy tissue samples from probable CAPMI cases for verification of definitive invasion of fungal agents in tissue, reflecting the current standard of care in Iran. These were among the main limitations of our study.

## Conclusion

Although the current guidelines are in favor of appropriate administration of antifungal treatment in CAPMI cases, the absence of appropriate antifungal treatment highlights that CAPMI is a neglected complication among mechanically ventilated COVID-19 patients admitted to ICUs in our study. Finally, CAPMI can be caused by species other than *Aspergillus*.

## Data Availability Statement

The raw data supporting the conclusions of this article will be made available by the authors, without undue reservation.

## Ethics Statement

This study was approved by the ethics committee of the Mazandaran University of Medical Sciences (Code: IR.MAZUMS.REC.1399.7697). The patients/participants provided their written informed consent to participate in this study.

## Author Contributions

MTH, LD, DSP, MH, WP, and AA were involved in the concept and design of the study. MG, MM, MA, IH, RM, SM, JC, WF, WL, and MHN were involved in the acquisition, analysis, and/or interpretation of the data. All authors participated in drafting the manuscript, its critical revisions for important intellectual content, and approved the final submitted article.

## Conflict of Interest

DSP received grant and contract funds from Merck, Regeneron, and Pfizer for COVID-19-related research programs. MHN received CDC-sponsored grant, CAPA. MH received research funding from Agile AS and Pfizer. The remaining authors declare that the research was conducted in the absence of any commercial or financial relationships that could be construed as a potential conflict of interest.
